# The Vaginal Microenvironment: The Physiologic Role of *Lactobacilli*

**DOI:** 10.3389/fmed.2018.00181

**Published:** 2018-06-13

**Authors:** Emmanuel Amabebe, Dilly O. C. Anumba

**Affiliations:** Academic Unit of Reproductive and Developmental Medicine, University of Sheffield, Sheffield, United Kingdom

**Keywords:** *Lactobacillus*, lactic acid, glycogen, estrogen, vaginal microbiota, bacterial vaginosis, preterm birth

## Abstract

In addition to being a passage for sperm, menstruum, and the baby, the human vagina and its microbiota can influence conception, pregnancy, the mode and timing of delivery, and the risk of acquiring sexually transmitted infections. The physiological status of the vaginal milieu is important for the wellbeing of the host as well as for successful reproduction. High estrogen states, as seen during puberty and pregnancy, promote the preservation of a homeostatic (eubiotic) vaginal microenvironment by stimulating the maturation and proliferation of vaginal epithelial cells and the accumulation of glycogen. A glycogen-rich vaginal milieu is a haven for the proliferation of *Lactobacilli* facilitated by the production of lactic acid and decreased pH. *Lactobacilli* and their antimicrobial and anti-inflammatory products along with components of the epithelial mucosal barrier provide an effective first line defense against invading pathogens including bacterial vaginosis, aerobic vaginitis-associated bacteria, viruses, fungi and protozoa. An optimal host-microbial interaction is required for the maintenance of eubiosis and vaginal health. This review explores the composition, function and adaptive mechanisms of the vaginal microbiome in health and those disease states in which there is a breach in the host-microbial relationship. The potential impact of vaginal dysbiosis on reproduction is also outlined.

## Introduction

**Figure 1:**
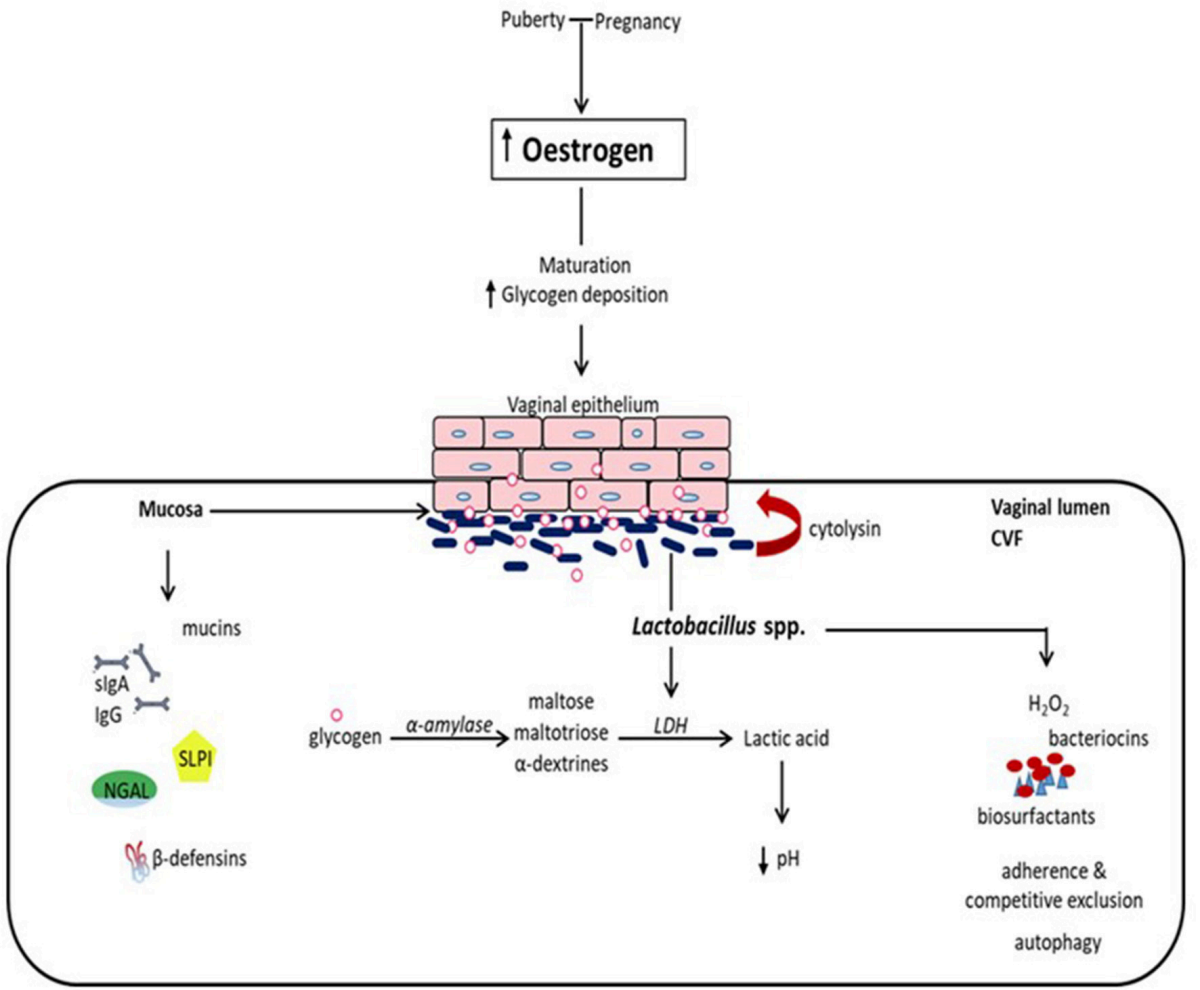
Eubiotic effect of estrogen and *Lactobacillus* species in the vaginal milieu. At puberty and during pregnancy, elevated levels of estrogen promote the maturation of and deposition of glycogen in vaginal epithelial cells. Glycogen from exfoliated and lysed epithelial cells is catabolized by α-amylase in the vaginal lumen to smaller polymers that are subsequently metabolized to lactic acid by *Lactobacillus* spp. Lactic acid and cytolysin produced by *Lactobacilli* stimulate the dissolution of epithelial cells by lysis and enhance the availability of glycogen. Lactic acid acidifies the vaginal milieu favoring the proliferation of *Lactobacilli* and inhibiting the growth of infection-associated organisms. This is reinforced by *Lactobacilli* through the production of hydrogen peroxide (H_2_O_2_), bacteriocins and biosurfactants, as well as the inhibition of the physical attachment of pathogens to the epithelium by competitive exclusion and the promotion of the engulfment and degradation of infected epithelial cells (autophagy). Additionally, there is concomitant production of mucins, immunoglobulins (secretory IgA and IgG), secretory leucocyte protease inhibitor (SLPI), neutrophil gelatinase-associated lipocalin (NGAL), and β-defensins, and other antimicrobial proteins, which all together provide a formidable first line of defense against infection. (*CVF*, cervicovaginal fluid; *LDH*, lactate dehydrogenase).

The vaginal mucosal ecosystem is comprised of a stratified squamous non-keratinized epithelium overlaid by a mucosal layer continuously lubricated by cervicovaginal fluid (CVF). Together, these form a daunting physical and biochemical barrier against extraneous invading organisms. Apart from being an acidic medium containing an assortment of antimicrobial molecules including antibodies (IgA and IgG), mucins, β-defensins, secretory leucocyte protease inhibitor (SLPI), neutrophil gelatinase-associated lipocalin (NGAL), surfactant protein etc., CVF also facilitates the confinement of exogenous organisms (summarized in Figure 1) (1–3).

The vagina also harbors numerous microorganisms (the “microbiota”), that exist (in conjunction with their genes and products) in a regulated mutualistic relationship with the host (the “microbiome”) (4). Some of these microorganisms such as *Lactobacillus* species reinforce the defense against invasion and colonization by opportunistic pathogens. The composition of the vaginal microbiota/microbiome is dynamic and undergoes changes corresponding with hormonal fluctuations throughout the woman's reproductive life, i.e., from puberty to menopause, and during pregnancy (5).

The normal physiological vaginal microbiota was initially described in 1892 by Albert Döderlein as homogenous, consisting of only Gram-positive bacilli (Doderlein's bacilli) (6), believed to originate from the gut and currently known to be a part of the genus *Lactobacillus* (7). The evolution of this unique vaginal microbiome is supported by two evolutionary hypotheses: the “disease risk hypothesis” (8, 9), and the “obstetric protection hypothesis” (9), which suggest that the human vagina is selectively dominated by protective *Lactobacillus* species because humans are more susceptible to sexually transmitted diseases; and also at higher risk of pregnancy and parturition-associated microbial complications (8–10).

A number of protective *Lactobacillus* species dominates the healthy vaginal microbiota in most reproductive-age women. Recent advances in DNA sequencing techniques have revealed that the dominant *Lactobacillus* species in the vaginal microbiota include *L. crispatus, L. gasseri, L. iners*, and *L. jensenii*, while other anaerobes including *Gardnerella, Atopobium, Mobiluncus, Prevotella, Streptococcus, Ureaplasma, Megasphaera* etc. able to cause infections such as bacterial vaginosis (BV) are kept dormant by the protective action of lactobacilli. These high-resolution techniques have enabled the classification of the vaginal microbiota into five community state types (CSTs) with CSTI, II, III and V dominated by *L. crispatus, L. gasseri, L. iners*, and *L. jensenii* respectively, while CSTIV is dominated by mixed anaerobes similar to those found in BV (5, 11).

The prevalence of these organisms in the vaginal microbiota vary in different race/ethnic groups and biogeographical locations, with Blacks and Hispanics harboring more anaerobic bacterial species (CSTIV) and showing higher vaginal pH in the presence or absence of clinical infection (11, 12). Differences in prevalence are also related to lifestyle differences (11) and gene-environment interactions (12). Unlike other body viscera such as the gut, increased diversity of the vaginal microbiota is linked to increased susceptibility to disease and negative reproductive outcomes (5, 13).

In addition to epithelial cells and microbiota, the vagina also contains immune-related cells (such as neutrophils, macrophages, T and B cells, natural killer (NK) cells, etc.) (14) and specialized receptors, e.g., toll-like receptors (TLRs) and nod-like receptors (NLRs), with which it recognizes the presence of pathogenic microbial species (15). Microbial stimulation through ligands such as lipopolysaccharide (LPS) that stimulates TLR-4 triggers the release of cytokines and chemokines such IL-1β, IL-6, IL-8, and tumor necrosis factor-α (TNF-α). This release is activated and regulated via a nuclear factor-κB (NF-κB)-mitogen-activated protein kinase (MAPK) signaling pathway (15). Other immune factors including macrophages, NK cells, helper and cytotoxic T cells as wells as B-lymphocytes are subsequently recruited to mount appropriate immune responses. Such pathogen-stimulated inflammatory responses normally control infection but can in some instances breach the mucosal surface and facilitate transmission of some other infections such as HIV (1). Therefore, vaginal communities dominated by anaerobes are potentially associated with greater pro-inflammatory response than *L. crispatus, L. gasseri* or *L. jensenii* (3, 5). However, *Lactobacilli* and lactic acid via multiple mechanisms as discussed below promote antimicrobial defense without inducing immune-mediated inflammation unlike the pathogenic anaerobes (3).

## The effect of estrogen on the vaginal ecosystem

The prepubertal vaginal microbiome is dominated by anaerobes, *E. coli*, diptheroids and coagulase-negative *Staphylococci* and significantly lesser glycogen (5). At puberty, the rising levels of estrogen promote the maturation, proliferation and accumulation of glycogen in the vaginal epithelial cells. Glycogen is catabolized by human α-amylase to maltose, maltotriose and α-dextrines, which are then metabolized to lactic acid by *Lactobacillus* species (Figure 1). This creates an acidic environment (pH, 3.5–4.5) conducive for the growth of *Lactobacilli* at the expense of other anaerobic bacterial species (1, 5). *Lactobacilli* dominance decreases as estrogen levels decline following menopause (16), and increases with vaginal estrogen replacement therapy.

The vaginal microbiota in normal pregnancy is predominated by *Lactobacilli* and is more stable than that in the non-pregnant state (12, 17, 18). This can be explained by the high level of estrogen during pregnancy resulting in increased vaginal glycogen deposition which enhances the proliferation of *Lactobacilli*-dominated vaginal microbiota (2). Also, studies have shown that menstruation significantly reversibly alters the vaginal microbial diversity, with about a 100-fold decrease in *L. crispatus* and increase in *L. iners, G. vaginalis, P. bivia*, and *A. vaginae* (19, 20). In essence, the normal acidic vaginal pH in reproductive-age women is driven by estrogen, glycogen, and *Lactobacilli* (1, 21–23).

An intriguing direct relationship between vaginal ostrogenization and candidiasis in postmenopausal women has also been reported (24). After menopause, estrogen-induced vaginal epithelial glycogen accumulation is associated with increased infection by *Candida albicans* that has glycogen as a major substrate. Contrastingly, in premenopausal women the activity of α-amylase that correlates with D- (but not L-) lactic acid and production of SLPI, NGAL, hyaluronidase-1 and matrix metalloproteinase (MMP)-8, was decreased in women infected with *C. albicans* (25). Increased glycogen availability secondary to exfoliation and lysis of glycogen-rich epithelial cells into the vaginal lumen by extracellular matrix degrading enzymes - hyaluronidase-1 and MMP-8, lactic acid and cytolysin could enhance α-amylase activity (25). This effect of vaginal ostrogenization, glycogen level and candidiasis in relation to menopausal status is likely to be of physiologic importance and necessitates further investigation.

## *Lactobacilli, sine qua non* of vaginal homeostasis

Vaginal lactic acid is predominantly of bacterial origin (26). Under the influence of estrogen, the vaginal epithelium produces <15% of L-lactic acid (26), while lactobacilli are the major source of both L- and D-lactic acid (27). Of the four most common vaginal *Lactobacillus* species, only *L. iners* lacks the ability to synthesize D-lactic acid and instead produces the L-isomer (3). Vaginal levels of α-amylase (produced by endocervical and fallopian tube cells) directly correlates with levels of D-lactic acid and other vaginal epithelial antimicrobial peptides such as SLPI and NGAL, but not with L-lactic acid (25). D-lactic acid is more protective against vaginal dysbiosis than L-lactic acid (27). Its levels are highest when *L. crispatus* is the dominant specie and lowest when *L. iners, Gardnerella* or *Streptococcus* predominate (27); and this partly accounts for the higher protection against urogenital infections (28, 29) and adverse reproductive outcomes (30) conferred by *L. crispatus* compared to *L. iners*. Lactic acid at physiological concentrations (e.g., 110 mM) acidifies vaginal secretions (to pH levels <4), enhances the protective activities of H_2_O_2_ and bacteriocins (31, 32), and inhibits opportunistic infections such as *G. vaginalis, Trichomonas vaginalis, Neisseria gonorrhoeae, Chlamydia trachomatis*, herpes simplex virus (HSV), human papillomavirus (HPV), HIV etc. (33). Specifically, distinct from its bactericidal activity D-lactic acid inhibits Chlamydia infection through a pH-dependent effect on the vaginal epithelial cells and microenvironment (34). This conclusion arose from the significantly greater protection against chlamydia provided by *L. crispatus, L. gasseri*, and *L. jensenii* (34) that produce more D-lactic acid than *L. iners* that produces predominantly L-lactic acid (35). Also, D-lactic acid prevents upper genital tract infection by modulating the L-lactic acid-induced production of extracellular matrix metalloproteinase inducer (EMMPRIN) from vaginal epithelial cells, and inhibiting the production of MMP-8 (27).

The precise mechanism of the bactericidal activity of *Lactobacillus* is unclear but there is evidence that it is mediated through the protonated forms of both D- and L-lactic acid and not the lactate anion (36). At a pH value < pKa 3.9, the protonated or uncharged form of lactic acid predominates. Because it is a weak acid, 50% of lactic acid dissociates into lactate anion and H^+^ at a pH of 3.86. Lactic acid in its protonated form is membrane-permeant and unlike the lactate anion, does not require the proton-linked monocarboxylate transporters or the lactate-binding GPR81 receptors to enter cells (37, 38). Lactic acid preferentially lyses bacteria other than *Lactobacillus* species (23, 36); and causes bacterial cell death by acidifying the cytosol, disrupting intracellular function (39), increasing the permeability of the cell membrane to H_2_O_2_, diacetyl etc., thereby potentiating the antimicrobial effect of other substances (40). The reduced antimicrobial activity of lactic acid and increased risk of infection associated with unprotected sexual intercourse and menstruation could be attributed to the increase in vaginal pH after deposition of seminal fluid and flow of menstruum, which leads to formation of more lactate anion that has less antimicrobial and immunomodulatory activities (33, 36).

Lactic acid also performs some immunomodulatory actions on the genital tract mucosa and other cell types (41, 42). Lactic acid in its protonated form creates an anti-inflammatory state by stimulating the production of large amounts of the anti-inflammatory cytokine IL-1RA, and inhibiting the production of inflammatory cytokines (IL-6 and TNFα) and chemokines (IL-8, RANTES and macrophage inflammatory protein-3α, MIP-3α) in the presence of TLR-2,−3,−4 agonists (41). Lactic acid also inhibits nuclear translocation and activation of the transcription factor NF-κB in peripheral blood mononuclear cells and monocytes/macrophages (43, 44). It is worth noting that both D- and L-lactic acid exercise these anti-inflammatory effects that are enhanced by low pH < 3.86 by directly acting on cervicovaginal epithelial cells (41, 42).

Also, both D- and L-lactic acid can enhance vaginal epithelial cell survival by facilitating the repair of damaged DNA through the inhibition of histone deacetylase activity leading to increased acetylation of histones on the surface of DNA (45, 46). This epigenetic regulation of gene expression (45) permits the transcription of genes that were previously blocked and possibly promotes the secretion of components of the antimicrobial innate immune system, such as NGAL from vaginal epithelial cells, that selectively prevent the growth of bacteria other than lactobacilli (3, 47). These observations show great promise for the use of lactic acid-containing microbicides for therapeutic restoration of vaginal homeostasis and prevention of STIs including HIV.

*Lactobacilli* (apart from *L. iners*) produce hydrogen peroxide (H_2_O_2_), which inhibits the growth of catalase-negative anaerobic organisms by production of hydroxyl free radicals (48, 49). They can also bind to the surface of vaginal epithelium and competitively prevent other microbes from attaching to and infecting the cells. *Lactobacilli* can produce other antimicrobial peptides such as bacteriocins, bacteriocin-like substances and biosurfactants; and promote autophagy (engulfment and degradation) of intracellular bacteria, viruses and protozoa (Figure 1) (27). Hence, through these mechanisms, lactobacilli inhibit the growth of other potentially pathogenic endogenous vaginal bacteria and prevent the acquisition of exogenous bacteria. For these reasons a lactobacilli-dominated vaginal microbiota has been described as healthy and necessary for the overall wellbeing of the woman. However, it is important to note that about 25% of women ostensibly maintain healthy vaginal microbiota without lactobacilli dominance. These women have been found to harbor other lactic acid producers such as *Atopobium, Megasphaera, Leptotrichia, Streptococcus*, and *Staphylococcus* (50, 51).

In addition, the degree of protection conferred on the vaginal ecosystem is dependent on the predominant *Lactobacillus* specie. For example, an *L. iners*-dominated vaginal microbiota is usually associated with dysbiosis and appears less stable and more prone to transition. In contrast *L. crispatus* that produces both D- and L-lactic acid is associated with increased vaginal community stability (less likely to transit to dysbiosis) and health (19, 52, 53). *L. iners* exhibits pathogenic propensity via its pore-forming cholesterol-dependent cytolysin (CDC, inerolysin) (54, 55). It has a small genome and is unable to produce D-lactic acid and H_2_O_2_ required to promote eubiosis, unlike the other *Lactobacillus* species (3, 13). Also, we recently observed that preponderance of *L. jensenii* (which produces only the D-Lactic acid and lesser protective capacity compared to *L. crispatus*) (27), was associated with decrease in lactate, succinate and increased risk of premature delivery (56).

## The influence of microbial activity on the vaginal mucosal barrier function

The mucosal surface of the vagina is an immunological and physical barrier that prevents potential pathogens from coming in contact with vaginal epithelial cells. It contains glycosylated mucus proteins (sialoglycoproteins) such as mucin that provide a dense lubricated physical barrier, which inhibits epithelial cell-pathogen contact; and secretory immunoglobulin A (sIgA) and IgG that recognize and neutralize antigenic microbial products. Anaerobes associated with vaginal infection such as *G. vaginalis* secrete sialidase that degrades mucus by cleaving sialic acid from the glycoproteins. Sialic acid is taken up and neutralized by *G. vaginalis* (foraging) to further circumvent the host response. A significant depletion of mucus sialic acids is seen in BV-infected women compared to their healthy counterparts with Lactobacillus-dominated microbiota (57). Degradation and depletion of the components of the mucosal protective barrier permits ascending upper genital tract infection. In addition, like *L. iners, G. vaginalis* also produce a CDC called vaginolysin with which it forms pores in the vaginal epithelium, which further obliterates the protective barrier. Contrarily, the protective function of the mucus layer is enhanced by *L. crispatus*-dominated microbiota thereby hindering the penetration of pathogens like HIV, whereas *L. iners*-dominated microbiota facilitates the penetration of HIV. Therefore, alterations in the composition of the vaginal microbial community significantly affects the integrity of the protective mucosal surface layer (5).

## Stress and vaginal health

The influence of stress on vaginal immunity has been the subject of much speculation. Immune response may be impaired by stress-related activation of the hypothalamic-pituitary adrenal (HPA) axis and secretion of corticotropin-releasing hormone (CRH) from the hypothalamus, which activates the release of cortisol from the adrenal cortex and noradrenaline from sympathetic nerve terminals (58). Cortisol inhibits the estrogen-associated vaginal epithelial maturation and accumulation of glycogen and consequently reduces lactobacilli dominance, while noradrenaline acts synergistically with immune mediators to potentiate the release of cytokines. The stress-induced increase in cortical hormones - cortisol and deoxycorticosterone - and the resultant decrease in lactobacilli abundance can worsen vulvovaginal symptoms of infection (59). Reduced vaginal epithelial glycogen decreases the production of lactic acid and loss of its anti-inflammatory activities. Hence, a dysbiotic vaginal flora is created characterized by a reduction or loss of lactobacillus dominance. Concomitant increase in noradrenaline potentiates the pro-inflammatory response and proliferation of pathogenic strict and facultative anaerobes as well as other STIs. Ultimately, stress exacerbates the susceptibility and severity of vaginal infection (3).

## Aberrant vaginal microbiota (dysbiosis)

The vaginal microbiota is a dynamic community of diverse bacterial species repeatedly subjected to both internal and external manipulative stimuli such as changes in sex hormone levels and stage of the menstrual cycle, sexual activity, antibiotic therapy and the use of oral contraceptives, vaginal douching, menopause, pregnancy, lactation, diabetes mellitus and stress. The composition of the vaginal microbiota is also determined by gene-environment interactions. Vaginal bacterial communities devoid of *Lactobacillus* dominance with higher pH and lower H_2_O_2_ have been observed to be normal in Black and Hispanic women (11, 52, 60, 61).

The most common vaginal infection in reproductive-aged women is bacterial vaginosis (BV). BV, with a prevalence rate of 5–70% (62), is characterized by a depletion of lactobacilli in favor of potentially pathogenic mixed anaerobes such as *Gardnerella, Atopobium, Mobiluncus, Prevotella, Streptococcus, Mycoplasma, Ureaplasma, Dialister, Bacteroides* etc. (63–65). It creates a more heterogeneous vaginal environment associated with decreased lactic acid levels, pH > 4.5, and high amounts of short chain fatty acids (SCFAs) such as acetate, butyrate, propionate and succinate produced by anaerobes. Although an overt inflammatory response is often not manifest, it has been associated with increased levels of immune mediators such as IL-1β, IL-2, IL-6, IL-8, IL-10, TNF-α, Interferon (IFN)-γ, RANTES etc. (1, 5), and decreased concentration of antimicrobial peptides like NGAL (47). BV is an enigmatic syndrome with unidentified etiology. Most BV-positive women are usually asymptomatic. However, symptoms could appear in the form of a non-itchy but irritating, creamy vaginal discharge with a fishy odor that may be more prominent after sexual intercourse and during menstruation.

The Amsel criteria are used to diagnose BV in most clinical settings. The criteria include the evaluation of vaginal acidity, the presence of vaginal discharge, the appearance of clue cells (desquamated vaginal epithelial cells studded with anaerobic bacteria), and a positive “whiff test” (a characteristic “fishy” odor perceived when 10% potassium hydroxide is added to a microscopic slide of vaginal discharge) (66). The most sensitive criteria are the vaginal pH (>4.5) and the detection of thin, homogenous, milky and adherent discharge (97%), when the criteria are assessed individually (67). However, the detection of discharge had a low specificity (26%) and positive predictive value (27%), while the criterion with the highest specificity was the presence of clue cells (86%). When combined together, the presence of at least three criteria significantly increases the likelihood of making an accurate diagnosis of BV, yielding a sensitivity and specificity of 97 and 90% respectively (67, 68).

In the research space, BV is commonly diagnosed with the Nugent scoring system (69). Although the diagnosis requires experienced laboratory staff to evaluate the slides, it is more objective and reliable and has a higher reproducibility and sensitivity compared to the Amsel criteria (70). It employs the Gram stain to microscopically identify a shift in vaginal microbiota from healthy lactobacilli dominance (Gram-positive rods) to an intermediate level of mixed Gram negative/variable microbiota (*Gardnerella* and *Bacteroides*), to absence of lactobacilli and predominance of Gram negative/variable rods or curved rods (*Mobiluncus*) (68, 69). A score of 0–3 is consistent with lactobacillus dominance and vaginal health, a score of 4–6 indicates an intermediate, mixed vaginal bacterial community, whereas >7 indicates BV infection. There is a good correlation between clinical features of BV and Gram stain scores (70).

BV is associated with increased risk of acquisition of STI such as *N. gonorrhoea, C. trachomatis, T. vaginalis*, HSV, HPV, and HIV, and other infections such as pelvic inflammatory disease, endometritis, chorioamnionitis, and amniotic fluid infection. In relation to pregnancy, BV appears to be associated with preterm premature rupture of membranes (PPROM), preterm labor (PTL) and preterm birth (PTB, i.e., delivery before 37 completed weeks of gestation) (62, 71–81).

Vaginal dysbiosis can also manifest as aerobic vaginitis (AV). It is an equally disruptive infection of the normal vaginal *Lactobacillus*-dominated microbiota but is characterized by overt inflammation, leukocyte and parabasal cell infiltration and proliferation of enteric aerobic bacterial organisms including *Escherichia coli, Enteroccoci, Staphylococcus aureus*, and group B *Streptococcus* (21, 82). It has been described as the aerobic equivalent of BV, due to decreased lactic acid concentration secondary to depleted *Lactobacillus* dominance. However, because anaerobes are absent, succinate concentration is low. AV has also been associated with STIs such as *C. trachomatis, N. gonorrhoeae*, and *T. vaginalis*. The clinical features of AV include: red vaginal mucosal inflammation, increased IL-1β and IL-6, vaginal pH > 6, itching and burning (pruritus), dyspareunia and yellowish sticky discharge devoid of fishy odor. AV is present in 2–25% of women and has been associated with severe adverse gynecological and obstetric outcomes including ascending genital infection/inflammation, PPROM, PTL and PTB (83, 84).

## Implications of abnormal vaginal microbiota for human pregnancy

The physiological status of the vaginal milieu is crucial not just to the general wellbeing of the host but also for conception and eventual success of pregnancy. The ability of *Lactobacillus* to preclude the invasion and colonization of the vaginal space by pathogens without triggering an overt inflammatory response is termed *Tolerance* and is particularly beneficial for reproduction (3). Apart from increasing the host's susceptibility to STIs and other gynecological conditions including cervical intraepithelial neoplasia and cervical cancer (85–87), a dysbiotic vaginal microenvironment with degraded Lactobacilli-mediated tolerance and anti-inflammatory mechanisms also influence the course and outcome of pregnancy. In essence, *Lactobacillus* spp. is invaluable to the preservation of a homeostatic vaginal milieu.

### Conception and miscarriage

Diminished vaginal *Lactobacillus* dominance has been linked to failure of *in vitro* fertilization (IVF) and miscarriage (88). Decreased conception rates and high rates of early pregnancy loss due to reduced concentration of H_2_O_2_-producing *Lactobacilli* and BV infection have been observed. The percentage of women who deliver a live birth after undergoing embryo transfer is greatly influenced by the microbial composition of the cervicovaginal space. Increased pregnancy loss at 10–16 weeks in spontaneous pregnancies (88) and early pregnancy loss (before 6 weeks) after IVF (89, 90) are associated with the presence of a dysbiotic vaginal microbiota during gestation. There is a high (30–40%) prevalence of abnormal vaginal microbiota in women undergoing IVF (89, 91, 92). Low pregnancy rate (18%) and increased pregnancy loss (up to 66%) was recorded in women undergoing IVF when *S. viridans* was present in the transfer catheter tip (92), while high live birth rates (up to 70%) and low or no pregnancy loss were observed with the presence of H_2_O_2_-producing *Lactobacillus* in the vagina and transfer catheter tip. An even lower clinical pregnancy rate (9%) associated with abnormal vaginal microbiota was observed in a recent study (93). The role of infection-induced inflammation of the endometrial lining and gestational tissues (chorioamniotic membranes and placenta) was implicated (88). However, this plausible association requires further investigation to unravel its pathogenesis in order to improve conception and live birth rates.

### Prematurity

**Figure 2:**
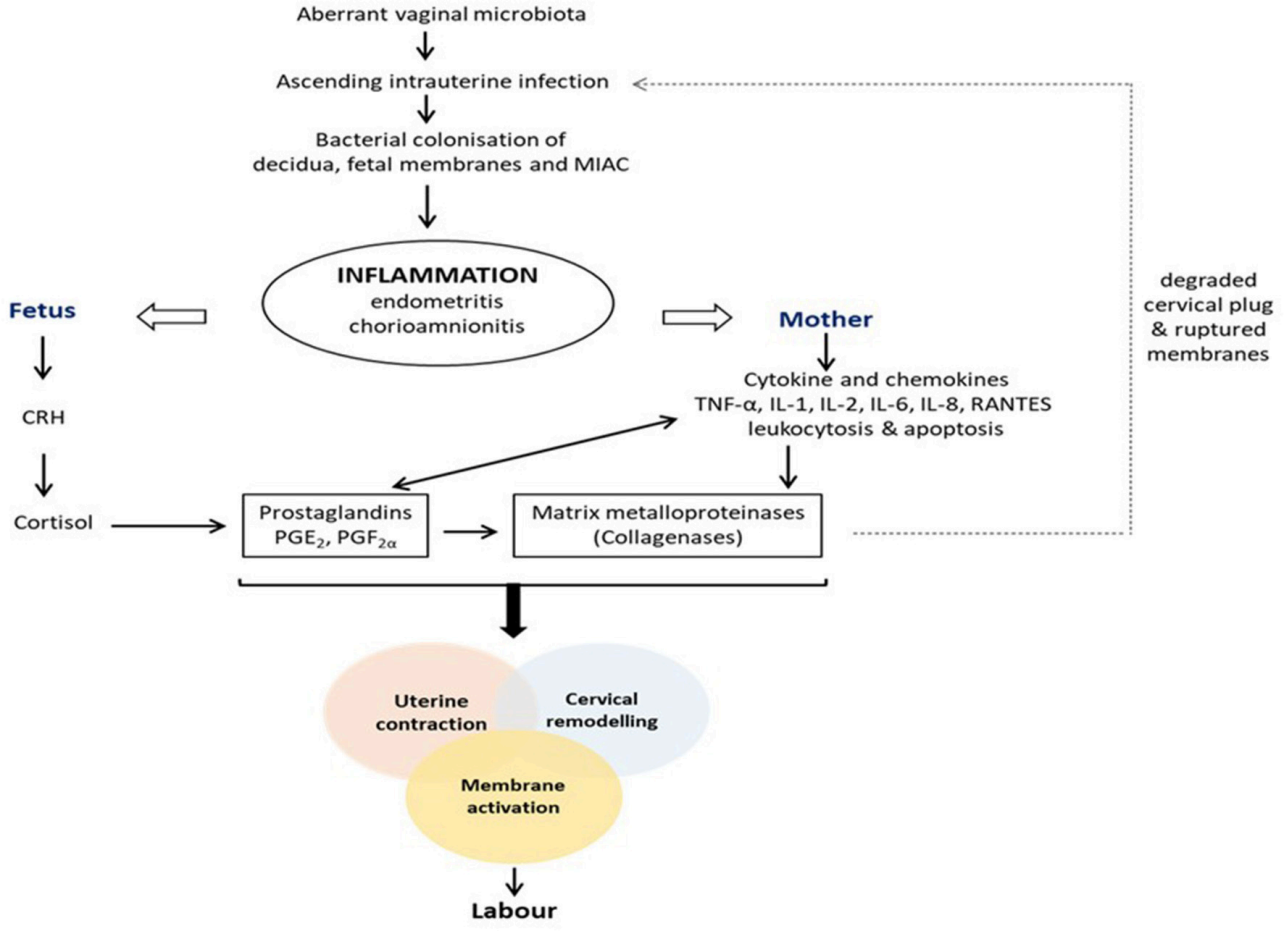
Pathogenesis of infection/inflammation-associated preterm labor and birth. Colonization of the female genital tract by pathogenic anaerobic bacteria due to an altered vaginal microbiota triggers a feed forward inflammatory response that ultimately stimulates the pathways to preterm labor and delivery, i.e., uterine contraction, cervical remodeling and membrane activation. CRH, corticotropin-releasing hormone; IL, interleukin; MIAC, microbial invasion of amniotic cavity; PGE_2_, prostaglandin E_2_; PGF_2α_, prostaglandin F_2α_; TNF, tumor necrosis factor.

Pregnancy is characterized by increased vaginal *Lactobacillus* dominance due to heightened estrogen-stimulated glycogen deposition and subsequent breakdown to lactic acid (Figure 1) (6, 12, 17). Also, tolerance mechanisms including lactic acid-induced autophagy of bacteria, viruses and protozoa, and lactobacilli inhibition of pro-inflammatory mediators are up-regulated during gestation (94, 95). Therefore, in the event of disturbance of this homeostatic vaginal host-microbial interaction during gestation (e.g., due to BV or AV), these protective mechanisms are diminished and invasion and colonization of the genital tract by pathogenic organisms can occur. These organisms can stimulate the degradation of the host mucosal epithelial barrier and cervical plug via the release of proteolytic enzymes such as elastase, mucinase, sialidase, protease, prolidase, and collagenases; ascend through the cervical canal and gain access into the uterine cavity and compromise the integrity of the fetal membranes (96–100). Ascending intrauterine infection earlier in gestation accounts for about 50% of spontaneous PTBs (2, 77), as does chorioamniotic-decidual inflammation (101). This occurs via the release of pro-inflammatory cytokines and chemokines (e.g., TNF-α and β, IL-1α and β, IL-2, IL-6, IL-8, RANTES etc.), prostaglandins (PGE_2_ and PGF_2α_), matrix metalloproteinases (MMPs- 1, 2, 3, 8, and 9), CRH, which trigger uterine contraction, membrane activation (i.e., separation of fetal membranes from the decidua) and cervical remodeling and effacement. An undesirable positive feed forward mechanism is established which culminates in PPROM, PTL and PTB (Figure 2) (49, 102). Central to these labor-associated processes (at both term and preterm) is the pleotropic transcription factor—NF-κB. It is activated by the recognition of microbial products (e.g., LPS) and non-microbial products by TLR/NLR, and regulates the expression of pro-inflammatory cytokines, prostaglandins and MMPs (15, 103).

Interestingly, D-lactic acid produced exclusively by *Lactobacillus* spp.—*L. crispatus, L. jensenii*, and *L. gasseri*—in the vagina, inhibits the synthesis of MMP-8 by modulating the action of EMMPRIN produced by vaginal epithelial cells (27). This prevents cervical tissue digestion and decreases the possibility of an ascending intrauterine infection and microbial invasion of the amniotic cavity (MIAC). Also, the protective function of the vaginal epithelial mucus layer is enhanced (5) and heightened autophagy activity (3) is observed when the vaginal microbiota is predominated by *L. crispatus*. An *L. crispatus*-dominated vaginal microbiome as seen in most healthy pregnancies is also inhibitory to *E. coli* colonization of the genital tract (104), and can prevent AV (83, 84). This is indicated to be via proteins produced by *L. crispatus* – S-layer, bacterial surface layer and cell separation proteins; and *L. jensenii* – adhesion exoprotein (105). In contrast, a low *E. coli* inhibitory activity was observed in women with *L. iners* dominance irrespective of pregnancy status (104), as this does not produce D-lactic acid. However, this observation still requires further investigation because in African women in whom the relative abundance of *L. crispatus* is low (11, 104), *E. coli* inhibitory activity is indicative of inflammation and increased susceptibility to HIV (106, 107). It is also worth noting that even in healthy women, there are ethnicity-dependent significant variations in the vaginal microbiome (108). Additionally, *L. crispatus* and *L. jensenii* inhibit the expression of pro-inflammatory mediators in the presence of TLR agonists (1). However, the mechanism through which this is achieved is unclear and the implications for reproductive outcomes may differ between these two species. Indeed, we recently observed an association between *L. jensenii* dominance, decreased lactate and succinate, and spontaneous PTB, while *L. crispatus* and *L. gasseri* were associated with elevated lactate and succinate, and delivery at term (56). We have also demonstrated a decrease in IL-8 and acetate levels (a marker of vaginal dysbiosis and imminent PTB) (1, 109–111) when *L. crispatus* is co-cultured with either *G. vaginalis* or *M. curtisii* (112).

A plausible explanation for these species-dependent varied outcomes could be the differential expression of lactic acid isomers by *L. crispatus, L. jensenii*, and *L. iners* in synergy with vaginal epithelial cells as described in earlier sections of this article. In addition to acidifying the vaginal milieu and inhibiting the growth of pathogenic anaerobes, both L- and D-lactic acid isomers (protonated form) exhibit anti-inflammatory properties by stimulating increased levels of IL-1RA without a concomitant increase in IL-1β, IL-6, IL-8, TNFα, RANTES, and MIP-3 α (41). They also attenuate the production of pro-inflammatory mediators stimulated by TLR agonist (41, 113, 114). Though both isomers exhibit similar virucidal and bactericidal activity against HIV-1 and BV-associated bacteria respectively (41, 42), D-lactic acid shows superior microbicidal capacity than the L-isomer (27, 115), and has been advocated as a prebiotic in the treatment of BV and prevention of PTB (2). The superior protective capacity of *L. crispatus* is attributable to its ability to produce both isomers of lactic acid. Taken together, the protective functions of *L. crispatus* and/or its products could be annexed through more comprehensive studies at different climates to develop therapeutic strategies for maintaining vaginal health and improving reproductive outcomes.

## Conclusion

A healthy vaginal milieu requires an optimum balance of the host-microbial interaction despite multiple and sometimes inevitable internal and external stimulations it receives. Alteration of this homeostatic state, perhaps due to failure of the host adaptive responses results in dysbiosis with reproductive consequences if left unattended. These manifest as decreased conception rates especially with assisted reproductive technology, early pregnancy loss, and premature labor and delivery. Though dysbiotic conditions such as BV also increase the rate of PTB up to 7-fold, especially when diagnosed before 16 weeks of gestation (70), most BV-infected pregnant women do not deliver prematurely (116). Also, a considerable proportion of women with inflammation of the gestational tissues who deliver preterm do not have BV (100). There is a growing body of evidence suggesting that it is the relative quantities (abundance) of bacteria, rather than just their presence (64, 117), and the unique host immune response to the infectious stimuli, that are associated with increased risk of infection and PTB (64, 118). Host differences in distribution of *Lactobacillus* spp. innate and adaptive immunity, volume and composition of vaginal fluid, expression of epithelia cells surface ligands, as well as other human behaviors and practices (such as smoking, sexual intercourse, douching, contraceptive and antibiotics use etc.) have been implicated in the variations in the composition of the “normal” vaginal microbiota. Risk of infection and PTB is modified by variations in maternal/fetal genetic composition and epigenetics, which ultimately determines the intensity of immune response to altered vaginal microbiota during gestation (119–125). These discrepancies have created a huge burden on the development of effective preventive and therapeutic strategies to improved women's health. As inflammation may continue optimally even after the stimuli (e.g., bacterial infection) has been treated, dual administration (cotreatment) of antibiotics and anti-inflammatory agents such as TNF biologics, CSAIDs – cytokine suppressive anti-inflammatory drugs (NF-κB and MAPK inhibitors) (126–128), PDE inhibitors, glucocorticoids, NSAIDs (15, 129, 130) should be explored.

## Author contributions

This review was conceived, conducted, written and reviewed by both EA and DA. Both authors approved the final manuscript.

### Conflict of interest statement

The authors declare that the research was conducted in the absence of any commercial or financial relationships that could be construed as a potential conflict of interest.

## Abbreviations

CDC: cholesterol-dependent cytolysin
CRH: corticotropin-releasing hormone
CSAIDs: cytokine suppressive anti-inflammatory drugs
CVF: cervicovaginal fluid
EMMPRIN: extracellular matrix metalloproteinase inducer
HIV: human immunodeficiency virus
HPA: hypothalamic-pituitary axis
HPV: human papillomavirus
HSV: herpes simplex virus
IL: interleukin
LPS: lipopolysaccharide
MAPK: mitogen-activated protein kinase
MIAC: microbial invasion of the amniotic cavity
MIP: macrophage inflammatory protein
MMP: matrix metalloproteinase
NF-κB: nuclear factor-κB
NGAL: neutrophil gelatinase-associated lipocalin
NK: natural killer
NLR: nod-like receptor
NSAIDs: non-steroidal anti-inflammatory drugs
PDE: phosphodiesterase
PGE_2_: prostaglandin E_2_
PGF_2α_: prostaglandin F_2α_
PROM: premature rupture of membranes
PRRs: pattern recognition receptors
PTB: preterm birth
PTL: preterm labor
RANTES: regulated on activation normal T cell expressed and secreted
SLPI: secretory leucocyte protease inhibitor
TLR: toll-like receptor
TNF: tumor necrosis factor.
